# Hexa-arginine enhanced uptake and residualization of selective high affinity ligands by Raji lymphoma cells

**DOI:** 10.1186/1476-4598-8-25

**Published:** 2009-04-22

**Authors:** Rod Balhorn, Saphon Hok, Sally DeNardo, Arutselvan Natarajan, Gary Mirick, Michele Corzett, Gerald DeNardo

**Affiliations:** 1 University of California, Department of Applied Science, Hertz Hall, PO Box 751, Livermore, CA 94551, USA; 2 Lawrence Livermore National Laboratories, Livermore, California 94551, USA; 3 University of California, Davis Medical Center, Sacramento, California 95816, USA

## Abstract

**Background:**

A variety of arginine-rich peptide sequences similar to those found in viral proteins have been conjugated to other molecules to facilitate their transport into the cytoplasm and nucleus of targeted cells. The selective high affinity ligand (SHAL) (DvLPBaPPP)_2_LLDo, which was developed to bind only to cells expressing HLA-DR10, has been conjugated to one of these peptide transduction domains, hexa-arginine, to assess the impact of the peptide on SHAL uptake and internalization by Raji cells, a B-cell lymphoma.

**Results:**

An analog of the SHAL (DvLPBaPPP)_2_LLDo containing a hexa-arginine peptide was created by adding six D-arginine residues sequentially to a lysine inserted in the SHAL's linker. SHAL binding, internalization and residualization by Raji cells expressing HLA-DR10 were examined using whole cell binding assays and confocal microscopy. Raji cells were observed to bind two fold more ^111^In-labeled hexa-arginine SHAL analog than Raji cells treated with the parent SHAL. Three fold more hexa-arginine SHAL remained associated with the Raji cells after washing, suggesting that the peptide also enhanced residualization of the ^111^In transported into cells. Confocal microscopy showed both SHALs localized in the cytoplasm of Raji cells, whereas a fraction of the hexa-arginine SHAL localized in the nucleus.

**Conclusion:**

The incorporation of a hexa-D-arginine peptide into the linker of the SHAL (DvLPBaPPP)_2_LLDo enhanced both the uptake and residualization of the SHAL analog by Raji cells. In contrast to the abundant cell surface binding observed with Lym-1 antibody, the majority of (DvLPBaPPP)_2_LArg6AcLLDo and the parent SHAL were internalized. Some of the internalized hexa-arginine SHAL analog was also associated with the nucleus. These results demonstrate that several important SHAL properties, including uptake, internalization, retention and possibly intracellular distribution, can be enhanced or modified by conjugating the SHALs to a short polypeptide.

## Background

Several strategies have been used to selectively deliver toxic chemicals or radiation to cancer cells [1,2], for gene therapy [3,4] or as tools for transfecting cells [5] and silencing genes [6]. Some of the earliest approaches used to enhance the cellular uptake of therapeutics and other molecules (fluorescent dyes, enzymes, antibodies and other proteins) involved introducing the molecules into liposomes or micelles [7,8]. Such constructs have been shown to fuse with the cell's membrane, introducing the contents inside the cell or transferring the lipid-bound components into the cell's membrane. Another highly successful approach has been to develop antibodies that target cell-specific membrane proteins and to use these antibodies to deliver radionuclides or other cytotoxic molecules to the surface of a specific population of cells [9-11]. More recently, intracellular delivery has been accomplished by attaching the molecules to be transported to naturally occurring transmembrane "shuttles", peptides or proteins that readily pass through cellular membranes. One of the more successful shuttles is a nuclear localization signal peptide derived from the SV40 T antigen [12]. This sequence, other peptide sequences derived from the transduction domain of the HIV-1 protein Tat [13,14], penetratin [15], and intact proteins such as the herpes virus protein VP22 [16] and anti-DNA antibodies [17] are currently being used to facilitate the transport of liposomes, viruses, enzymes, antibodies and a variety of other proteins into cells. Considerable success has also been achieved using synthetic cationic peptide transporters such as oligoarginine [18-21], lactosylated poly-L-lysine [22] and short peptide sequences selected from phage display libraries [23] that exhibit sequence similarities to know peptide shuttles.

Recently, several small molecule antibody mimics that show promise as targeting agents for cancer imaging or therapy have been synthesized [24-28]. In addition to exhibiting selectivities and affinities (nM to pM) similar to antibodies, these molecules have the potential to minimize some of the difficulties associated with the use of protein-based drug delivery systems. They retain the more desirable pharmacokinetic properties of small molecules, are less likely to be immunogenic, may prove stable enough for oral delivery, and the costs associated with producing the drug can be reduced significantly. The SHAL family of antibody mimics can also be easily modified to carry radioactive metals, a variety of tags that enable their use as imaging agents, and other small molecules (e.g. toxins or inhibitors). Another potentially useful modification includes alterations that facilitate uptake and internalization of the SHAL by the targeted cell, which would be expected to both increase tumor residence time and deliver the SHAL into an environment (the cytoplasm or nucleus) where it could cause additional damage.

Working with a SHAL developed previously for targeting HLA-DR10, an abundant cell surface receptor over-expressed on B-cell malignancies, we synthesized a peptide analog to the SHAL by conjugating it to hexa-arginine, a peptide that has been demonstrated previously to facilitate the transport of proteins and nucleic acids into cells. Binding studies conducted with the SHAL and its hexa-arginine analog *in vitro *using HLA-DR10 expressing Raji cells show that the hexa-arginine sequence changed the SHALs properties significantly, enhancing both SHAL internalization and radionuclide residualization.

## Results

### SHAL Design and Synthesis

**Figure 1:**
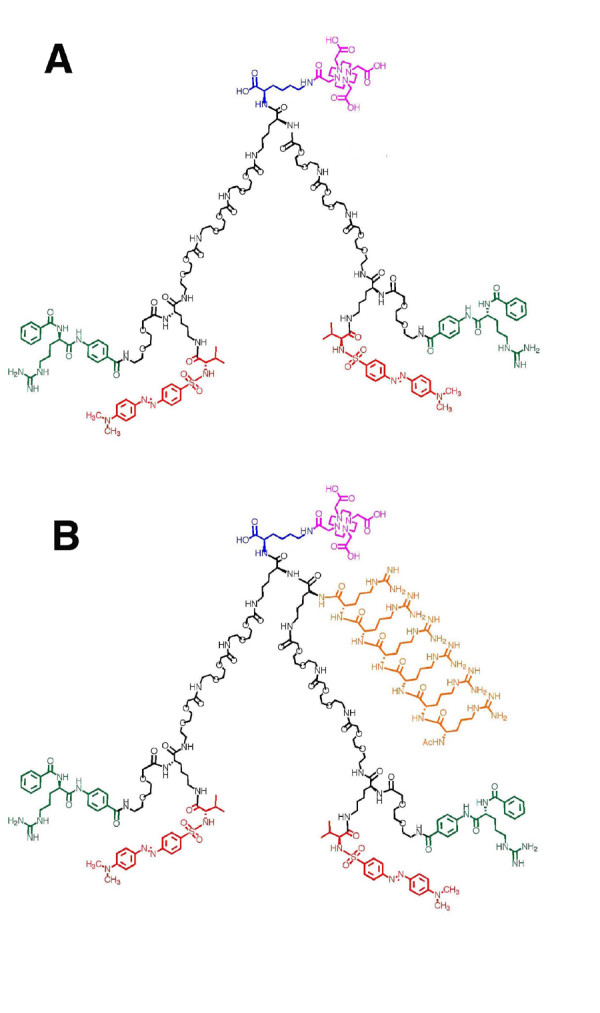
**Chemical structures of the dimeric SHAL (DvLPBaPPP)_2_LLDo (A) and the hexa-arginine analog (DvLPBaPPP)_2_LArg_6_AcLLDo (B)**.

Two forms of the free amine SHAL, (DvLPBaPPP)_2_LLA, and the hexa-arginine analog, (DvLPBaPPP)_2_LArg_6_AcLLA, were synthesized in multi-milligram amounts and purified by high performance liquid chromatography (HPLC). A biotin was attached to the ε-amino group of the terminal amine (A) on both (DvLPBaPPP)_2_LLA and (DvLPBaPPP)_2_LArg_6_AcLLA to produce biotinylated forms for use in cell and protein binding experiments. 1,4,7,10-tetraazacyclododecane-1,4,7,10-tetraacetic acid (DOTA) was attached to both (DvLPBaPPP)_2_LLA and (DvLPBaPPP)_2_LArg_6_AcLLA at the same site to enable the SHALs to be labeled with ^111^In. The DOTA SHAL (DvLPBaPPP)_2_LLDo and the hexa-arginine SHAL analog (DvLPBaPPP)_2_LArg_6_AcLLDo (Figure 1) were labeled with ^111^In at high efficiency (>90%) with specific activities ranging from 70–85 μCi/μg SHAL. Analyses of the resulting radiolabeled SHAL by HPLC and cellulose acetate electrophoresis (CAE) showed the purity of the product to be greater than 90%. D-isomers of arginine incorporated during the synthesis of the hexa-arginine sequence in (DvLPBaPPP)_2_LArg_6_AcLLDo were used to minimize the proteolytic susceptibility of the peptide. While more detailed experiments need to be carried out to adequately assess the stability of the SHAL *in vivo*, data obtained from one preliminary CAE experiment showed no evidence of degradation when the hexa-D-arginine SHAL analog was incubated in human plasma at 37°C for 24 hrs (data not shown).

### SHAL Affinity for HLA-DR10 Protein

Surface Plasmon resonance binding studies were conducted with both SHALs to estimate and compare the affinity of the two SHALs for isolated HLA-DR10 protein. In a series of kinetic experiments in which biotinylated versions of the SHALs were immobilized on the surface of a streptavidin chip, the parent SHAL (DvLPBaPPP)_2_LLDo was observed to bind to HLA-DR10 with a Kd ~21 nM. A similar Kd, ~34 nM, was obtained for the hexa-arginine containing analog (DvLPBaPPP)_2_LArg_6_AcLLDo.

### Analysis of SHAL Uptake by Raji Cells Expressing HLA-DR10

*In vitro *cell binding experiments were conducted using ^111^In-labeled parent SHAL and the hexa-arginine SHAL analog to quantify SHAL uptake and to evaluate the effect of adding the hexa-arginine tag. Uptake was assessed using Raji cells, a lymphoma cell line expressing HLA-DR10. Aliquots containing 10^6 ^cells were incubated with increasing amounts of SHAL containing ^111^In labeled SHAL as a tracer, and cell-associated ^111^In was measured before and after washing the cell pellets.

**Figure 2:**
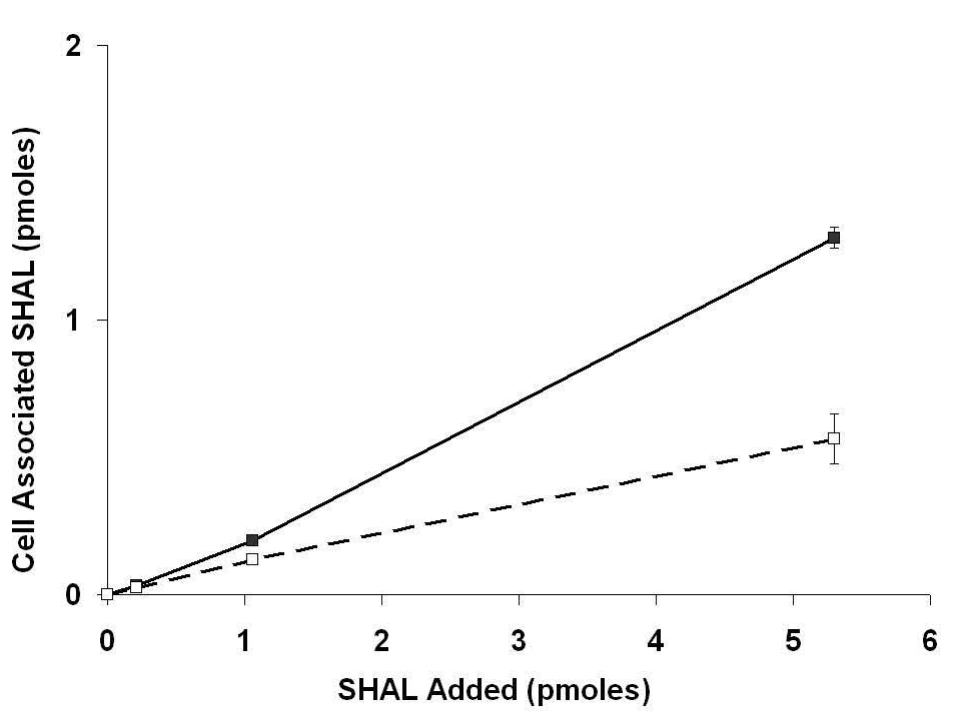
**Binding of ^111^In radiolabeled SHAL (DvLPBaPPP)_2_LLDo and its hexa-arginine analog (DvLPBaPPP)_2_LArg_6_AcLLDo to Raji cells**. Total ^111^In- (DvLPBaPPP)_2_LArg_6_AcLLDo bound to Raji cells (unwashed), solid squares; total ^111^In-(DvLPBaPPP)_2_LLDo bound to Raji cells (unwashed), open squares. Cell pellets containing 10^6 ^cells were resuspended in 150 μl 5%BSA/PBS buffer containing 0–25 ng of ^111^In labeled SHAL and incubated at RT for 1 hour. The samples were centrifuged to separate the cells from the supernatant and both were counted in a calibrated gamma well counter to quantify bound and unbound SHAL. Error bars are included for each data point, but in the majority of the cases the error is smaller than the data point and the error bar is not visible.

Analyses of the unwashed cell pellets showed that both the parent SHAL and the hexa-arginine SHAL are bound by Raji cells. Cell associated SHAL increased linearly with increasing SHAL concentration in the media for both SHALs (Figure 2), and the amount of bound SHAL showed no evidence of reaching saturation over the range of SHAL concentration tested. Raji cells treated with the hexa-arginine SHAL, in contrast to those treated with the parent SHAL, bound twice as much SHAL (Table 1). A larger proportion of the hexa-arginine SHAL (67%) was also retained by the cells after washing when compared to the parent SHAL (~46%), leading to a final hexa-arginine SHAL content three times that of its parent.

**Table 1:** Retention (residualization) of bound SHAL by Raji cells.

	**pmoles SHAL Bound/10^6 ^cells**		**Percent SHAL Retained**
**SHAL**	**Unwashed**	**Washed**	

(DvLPBaPPP)_2_LLDo	0.568 ± 0.091	0.263 ± 0.000	46

(DvLPBaPPP)_2_LArg_6_AcLLDo	1.300 ± 0.038	0.876 ± 0.017	67

Identical samples of cells used in experiments shown in Figure 2 were incubated as described in the Materials and Methods with 5.3 pmoles of ^111^In-labeled (DvLPBaPPP)_2_LLDo or (DvLPBaPPP)_2_LArg_6_AcLLDo and then washed two times with BSA/buffer. The cell pellets were then counted to provide estimates of SHAL remaining bound after washing.

### SHAL Localization by 3-D Confocal Microscopy

**Figure 3:**
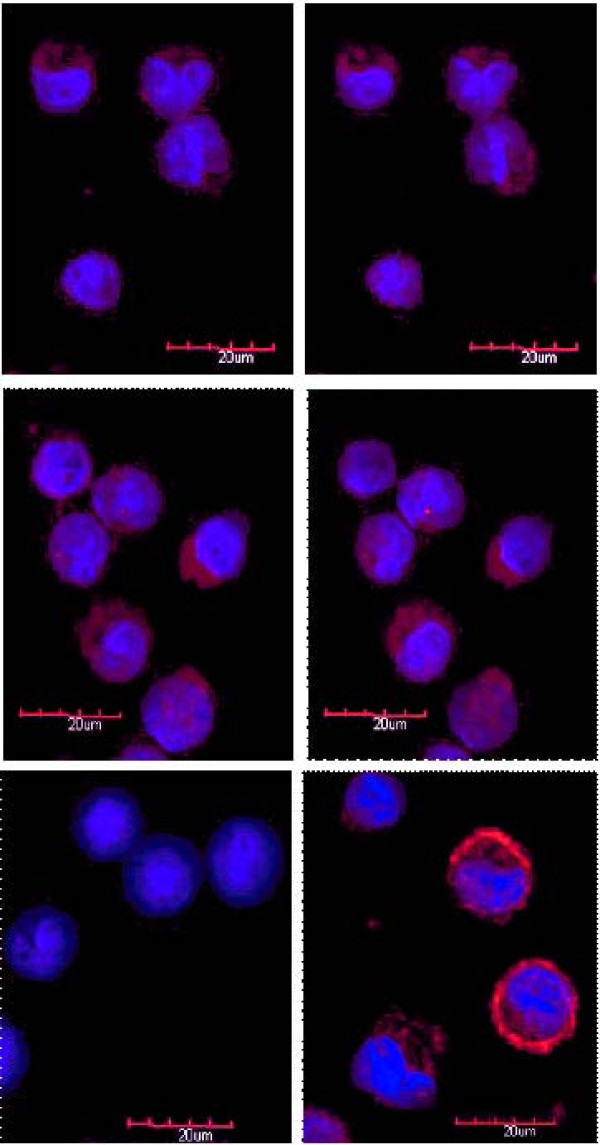
**Fluorescent 3-D confocal microscopy of parent SHAL (DvLPBaPPP)_2_LLDo (top row) binding to live Raji cells compared to the hexa-arginine analog (DvLPBaPPP)_2_LArg_6_AcLLDo (middle row)**. Two mid-cell focal planes within the Raji cells are shown (left to right). Jurkat's cells treated with (DvLPBaPPP)_2_LArg_6_AcLLDo (left panel, bottom row) show minimal SHAL uptake. Lym-1 (right panel, bottom row) exhibits primarily cell surface membrane binding to Raji cells. The parent SHAL shows intracellular binding, while the hexa-arginine analog demonstrates not only marked cytoplasmic binding but also intranuclear targeting. DAPI (blue) is used as the nuclear stain and AlexaFlor 610 (red) demonstrates the location of SHAL in these merged sequential laser images.

Fluorescence images collected at focal planes near the center of Raji cells treated with biotinylated forms of the parent and hexa-arginine SHALs for only an hour confirmed that both SHALs were taken up by Raji cells (Figure 3). In contrast to Lym-1 antibody, which binds to HLA-DR10 on the cell surface, the sectioned images taken from the center of the cells showed that both SHALs were localized inside Raji cells and distributed throughout the cytoplasm. Raji cells took up significantly more of the hexa-arginine SHAL than the parent SHAL, as evidenced by the more intense staining of the cytoplasm of cells treated with equivalent concentrations of the two SHALs. SHAL uptake was not observed in control Jurkat's cells (cells lacking HLA-DR10). A fraction of the hexa-arginine SHAL also appeared to be associated with the nucleus. Nuclear staining was not observed in cells treated with the parent SHAL.

## Discussion

Numerous cell penetrating peptides (CPPs) derived from viral and other proteins that traverse cell and nuclear membranes have been employed as shuttles to improve the efficiency of transport of liposomes, exogenous proteins and nucleic acids, and other molecules into the cytoplasm and nuclei of cells [13-23]. Studies characterizing the efficiency of internalization of different CPP sequences, all of which have a high content of arginine residues [29], have shown that arginine homopolymers containing as few as six arginine residues are highly effective in transporting small organic molecules [30,31] and large proteins into cells [32].

In an effort to develop SHALs that are more efficiently internalized and residualized by the cells they target, we synthesized a hexa-arginine conjugate of (DvLPBaPPP)_2_LLDo, a SHAL containing the two ligands dabsylvaline (Dv) and N-benzoyl-L-arginyl-4-amino benzoic acid (Ba) that had been shown previously to bind selectively to HLA-DR10 expressing cell lines [24]. Hexa-arginine was chosen as the first shuttle sequence to be tested for its ability to facilitate the transport of SHALs into cells because it could be conjugated to a dimeric SHAL without changing its molecular mass significantly, thereby preserving the desirable properties of the SHAL as a small molecule therapeutic. Surface Plasmon resonance experiments comparing the binding of the SHAL and the hexa-arginine SHAL analog to purified HLA-DR10 protein showed that the addition of the hexa-arginine peptide to the dimeric SHAL did not interfere with SHAL binding to the protein.

3-D Confocal microscopy experiments revealed that both the parent SHAL and its hexa- arginine analog were taken up and internalized by HLA-DR10 expressing Raji cells. SHAL uptake was not observed in Jurkat's cells, a cell line lacking HLA-DR10. Optical sections taken through Raji cells showed that the binding of the SHALs was not confined to the cell surface, as is characteristic of Lym-1 antibody binding. Mid-plane sections taken from cells treated with the SHALs showed the SHAL-associated fluorescence to be distributed throughout the interior of the cells. In some images, areas of high SHAL concentration within the cytoplasm occasionally appeared to be associated with small organelle-like structures. The cytoplasm-associated fluorescence was significantly higher in Raji cells treated with the hexa-arginine SHAL analog, suggesting the addition of the hexa-arginine peptide enhanced cell uptake of the SHAL.

Experiments comparing the binding of ^111^In-labeled (DvLPBaPPP)_2_LLDo and (DvLPBaPPP)_2_LArg_6_AcLLDo to live Raji cells confirmed that the hexa-arginine tag enhanced SHAL uptake. The presence of the tag also increased the amount of ^111^In-labeled SHAL that was retained by Raji cells. The amount of SHAL retained after washing did not reach saturation over the concentration range tested, suggesting that even higher concentrations of SHAL may be accumulated inside HLA-DR10 expressing cells than achieved in these experiments. At the highest concentration of hexa-arginine SHAL tested in the cell binding studies, the amount of residualized SHAL was equivalent to ~1.1 × 10^6 ^SHAL molecules per cell – the same number of HLA-DR10 molecules reported previously to be present on the surface of each Raji cell [33]. These results, together with the confocal images showing the majority of the hexa-arginine SHAL is internalized, indicate that a significant fraction of the SHAL may be bound to the pool of HLA-DR10 known to be present inside the cell.

The observed enhancement in residualization of ^111^In-labeled hexa-arginine SHAL by Raji cells and the potential association of a fraction of the ^111^In-label with the nucleus are also important because radioisotope internalization and residualization have been shown to be highly advantageous for cancer therapy [34-37]. Cancer therapeutics have been linked to a variety of radioisotopes that emit beta particles, alpha particles or Auger electrons. The range of beta emissions from isotopes routinely used in radioimmunotherapy, such as iodine-131, yttrium-90, and rhenium-188, extend for several millimeters, and therapeutics carrying these radionuclides create a "crossfire" [1,38] or "bystander" [39] effect destroying malignant cells to which the targeting agent is not directly bound. In this way, beta-emitters can potentially overcome resistance due to antigen-negative tumor cells. These characteristics make beta-particle therapy better suited for treating bulky tumors or large-volume disease. However, longer-ranged beta emissions can also destroy nearby normal cells.

The internalization of targeting agents such as the hexa-arginine SHAL (DvLPBaPPP)_2_LArg_6_AcLLDo can be exploited as a means of introducing Auger electron-emitting ^111^In into the cytoplasm and nucleus of cells where the Auger electrons have a very short, subcellular path length and high linear energy transfer [40-42]. The radiation absorbed dose to the nucleus has been estimated to be 2-fold and 35-fold greater when ^111^In decays in the nucleus compared to when decay occurs in the cytoplasm or on the cell surface, respectively [43,44]. These properties render ^111^In and other Auger electron-emitters highly cytotoxic and damaging to DNA when they decay in close proximity to the cell nucleus [45]. By coupling Auger emitters to highly selective, residualizing targeting agents that accumulate to high concentrations inside tumor cells, a very powerful class of therapeutics may be developed that are more effective in treating many types of metastatic cancer.

## Conclusion

The enhancement in hexa-arginine SHAL internalization by HLA-DR10 expressing lymphoma cells and the magnitude of the increase in SHAL residualization achieved by conjugating a hexa-arginine peptide to the SHAL are important because they show that small molecules such as SHALs can be designed to deliver radionuclides to malignant cells under conditions that lead to residualization of significant concentrations of radionuclide inside the cell. If additional studies confirm that hexa-arginine or other peptide SHAL analogs accumulate near or inside the nucleus, SHALs carrying Auger-emitting radionuclides may provide an alternative approach for increasing the therapeutic index achieved with SHALs beyond that attained by the accumulation of radionuclide-tagged targeting agents on the surface of the tumor cell. These results are also exciting because of the relevance of the SHAL-based approach to treating other forms of cancer. Internalizing SHALs targeting under-glycosylated MUC1, the androgen receptor and other tumor specific cell surface proteins that residualize the radioisotopes they carry could also be developed as small molecule therapeutics for a wide variety of other types of metastatic cancer.

## Methods

### SHAL Design, Synthesis, and Radiochemistry

#### Design

The process used to create a homology model for HLA-DR10, identify unique binding cavities within the Lym-1 epitope, select ligands that bind in these cavities, and create the (DvLPBaPPP)_2_LLDo SHAL has been reported previously [24]. A process for producing a hexa-arginine peptide analog of this parent SHAL, (DvLPBaPPP)_2_LArg_6_AcLLDo, was developed by modifying the synthesis to include the incorporation of an additional lysine residue into the middle of the linker connecting the two SHAL monomers and attaching an arginine hexapeptide to the free amine on this lysine.

#### Synthesis

The two dimeric SHALs (DvLPBaPPP)_2_LLA and (DvLPBaPPP)_2_LArg_6_AcLLA were synthesized on chlorotrityl chloride resin using orthogonally protected lysine (L) residues and miniPEGs (P) to link the two small ligands Dv and Ba as previously described for (DvLPBaPPP)_2_LLA [24,46]. To produce the amine derivative of the hexa-arginine SHAL (DvLPBaPPP)_2_LArg_6_AcLLA, a second Dde-D-Lys(Fmoc)-OH lysine residue was inserted into the linker during SHAL synthesis by performing two sequential Dde-D-Lys(Fmoc)-OH coupling steps. At the alpha position of the third lysine, six consecutive arginine residues were inserted by reacting the resin with Fmoc-D-Arg(Pbf)-OH six times. The sixth Arg residue was protected with an acetate (Ac) by reacting with acetic anhydride in N, N diisopropyl-ethylamine (DIEA)/dimethylformamide (DMF). The guanidinium groups on all six arginine residues remain protected with trifluoroacetic acid (TFA)-sensitive 2,2,4,6,7-Pentamethyldihydrobenzofuran-5-sulfonyl (Pbf) protecting groups throughout the rest of the synthesis. The remainder of the synthesis was then completed as described previously for (DvLPBaPPP)_2_LLA [24,46]. Analytical HPLC and electrospray ionization mass spectrometry (ESI-MS) were performed to confirm the purity and identity of the (DvLPBaPPP)_2_LLA and (DvLPBaPPP)_2_LArg_6_AcLLA free amine SHALs.

##### (DvLPBaPPP)_2_LLA

Starting with 50 mg (0.07 mmol) resin and 30 mg (0.07 mmol) Fmoc-D-Lys(Boc)-OH, 34 mg of (DvLPBaPPP)_2_LLA (Rt = 7.86 min, Waters Symmetry C18, 5 μm, 4.2 × 150 mm column, diode array detector with a linear gradient from 95% H_2_O, 1% TFA to 80% acetonitrile (MeCN), 1% TFA over 12 min) was isolated as a red solid after purification. ESI-MS: *m*/*z *calculated for C_150_H_224_N_34_O_41_S_2 _(M + 3H)^3+ ^1075.60, found 1075.62; calculated for (M + 4H)^4+ ^806.95, found 806.93; calculated for (M + 5H)^5+ ^645.76, found 645.68; calculated for (M + 6H)^6+ ^538.30, found 538.21.

##### (DvLPBaPPP)_2_LArg_6_AcLLA

81 mg of (DvLPBaPPP)_2_LArg_6_AcLLA (Rt = 8.30 min) starting from 90 mg (0.12 mmol) resin and 154 mg (0.29 mmol) Fmoc-D-Lys(Boc)-OH was isolated as a red solid after purification. ESI-MS: *m*/*z *calculated for C_194_H_310_N_60_O_49_S_2 _(M + 3H)^3+ ^1444.71, found 1444.65; calculated for (M + 4H)^4+ ^1083.76, found 1083.78; calculated for (M + 5H)^5+ ^867.23, found 867.18; calculated for (M + 6H)^6+ ^722.86, found 722.78; calculated for (M + 7H)^7+ ^619.74, found 619.62.

##### Attachment of DOTA to SHALs

The amine analog of the SHAL (DOTA-SHAL precursor with a free epsilon amine on the first lysine) was dissolved in 500 μl anhydrous DMF and 100 μl DIEA. The hexafluorophosphate (PF_6_) salt of DOTA N-hydroxysuccinimide (NHS) ester (933.36 g/mol, 1–1.5 equivalents) was added to the mixture as a solid. The mixture was nutated for 15 min and the reaction was monitored by analytical HPLC. Upon completion the reaction solution was diluted with 300 μl H_2_O and 300 μl MeCN (both containing 1% TFA) and HPLC purified using an 85% H_2_O (0.1% TFA) to 70% MeCN (0.1% TFA) gradient run over 25 min. The resulting purified DOTA-SHALs were lyophilized and subsequently analyzed by analytical HPLC (Waters Symmetry C18, 5 μm, 4.2 × 150 mm column, diode array detector) using a linear gradient from 95% H_2_O (1% TFA) to 80% MeCN (1% TFA) over 12 min) and characterized by ESI-MS.

##### (DvLPBaPPP)_2_LLDo

Reaction of the (DvLPBaPPP)_2_LLA amine SHAL (6.0 mg, 1.86 μmol) with DOTA NHS ester (2.0 mg, 2.14 μmol) gave 100% (Rt = 7.664 min) conversion by crude analytical HPLC and yielded (DvLPBaPPP)_2_LLDo (8.0 mg, red solid) after purification. ESI-MS: m/z calculated for C_166_H_250_N_38_O_48_S_2 _(M + 2H)^2+ ^1806.09, found 1806.22; calculated for (M + 3H)^3+ ^1204.40, found 1204.49; calculated for (M + 4H)^4+ ^903.55, found 903.61; calculated for (M + 5H)^5+ ^723.04, found 723.07; calculated for (M + 6H)^6+ ^602.70, found 602.64.

##### (DvLPBaPPP)_2_LArg_6_AcLLDo

Reaction of (DvLPBaPPP)_2_LArg_6_AcLLA amine SHAL (15.0 mg, 3.46 μmol) with DOTA NHS ester (5.0 mg, 5.36 μmol) gave 100% (Rt = 7.70 min) conversion by crude analytical HPLC and yielded (DvLPBaPPP)_2_LArg_6_AcLLDo (12.0 mg, red solid) after purification. ESI-MS: m/z calculated for C_210_H_336_N_64_O_56_S_2 _(M + 3H)^3+ ^1573.51, found 1573.54; calculated for (M + 4H)^4+ ^1180.38, found 1180.43; calculated for (M + 5H)^5+ ^944.51, found 944.52; calculated for (M + 6H)^6+ ^787.26, found 787.26; calculated for (M + 7H)^7+ ^674.94, found 674.88; calculated for (M + 8H)^8+ ^590.69, found 590.58.

#### Radiochemistry

As described previously [24,26], the DOTA-SHALs were labeled with carrier-free ^111^InCl_3 _(MDS Nordion, Vancouver, Canada) using the following method [26]. An aliquot of ^111^InCl_3 _(15–20 μl) was added to a solution of DOTA-SHAL (25–50 μg) in 0.1 M NH_4_OAc, pH 5.3 (50 μl); the final pH of the reaction mixture was adjusted to 6.5 by adding 4 M NH_4_OAc and the mixture was incubated for 1 h at 37°C, then 10–20 μl of 0.1 M ethylenediaminetetraacetic acid (EDTA) was added to sequester excess, free ^111^In^3+^. The radiolabeled product was purified using HPLC, followed by dialysis in phosphate-buffered saline (PBS) with a 1 kD cut off membrane. The purity of the ^111^In-labeled SHALs was determined by thin layer chromatography (TLC) (10% NH_4_OAc-MeOH 1:1), HPLC and cellulose acetate electrophoresis (CAE). CAE resolved ^111^In-DOTA-SHALs and ^111^In-EDTA; radioactive peaks were observed at 2.3–3.0 cm and > 6.5 cm, respectively. Similar results were observed in the TLC assay; ^111^In-DOTA-SHALs showed little migration from the point of application (R_F _= 0.25–0.3), whereas ^111^In-EDTA moved towards the solvent front (R_F _= 0.5). By HPLC, ^111^In-EDTA eluted at 2.5–3.0 ml and ^111^In-DOTA-SHALs at 9.5–10 ml. The ^111^In labeled SHALs were purified using RP-HPLC or a 1 kD dialysis membrane in PBS, and concentrated using a Savant Speedvac SC110 (Thermo Fisher Scientific, Inc, Waltham, MA, USA). Final radiochemical purity was determined using C18-RP-TLC (EM Science, DC-Plastikfolien kieselgel 60 F254, Cherry Hill, NJ), HPLC, and CAE. ^111^In-DOTA-SHAL product yields ranged from 70 – 90% and the purity of the product ranged from 90 – 95%. The final product was dissolved in 10% dimethylsulfoxide (DMSO) in PBS, and proved stable over 72 hours at room temperature.

### SHAL Binding to Isolated HLA-DR10 Protein

Protein binding experiments were conducted using surface plasma resonance on a Biacore 3000 (Biacore, Piscataway, NJ) at 25°C. A research grade streptavidin immobilized chip (SA chip, Biacore) was preconditioned and normalized according to the manufacturers instructions. Biotin labeled SHALs were dissolved in DMSO and diluted in 1.05× PBS (Biacore) to a final concentration of 1× PBS pH 7.4, 5% DMSO, to match the running buffer. These SHALs were injected over the flow cell to yield a surface density of 500–1000 RU (response units). Biotin (50 μM E-Z Link Amine-PEO2-Biotin, Pierce) was injected over all cells for 1 minute at 20 μl/min as a block to reduce non-specific binding. One flow cell was used as a reference cell and a different SHAL was immobilized on each of the three other cells.

Experiments measuring the binding of HLA-DR10 to the SHALs were carried out at a flow rate of 30 μl/minute in PBS pH7.4 running buffer using all 4 flow cells. HLA-DR10 isolated from Raji cells [47] was diluted in running buffer to a final concentration ranging from 10 nM to 1 μM, and a series of concentrations were run randomly in triplicate. Protein was injected for 3 minutes, allowed to dissociate for 5 minutes followed by regeneration of the surface using a 1 minute injection of 0.1% sodium dodecylsulfate (SDS) followed by a washing step with a 2 minute injection of running buffer. The data, which were double referenced by subtracting the blank reference surface and an average of 5 blank injections, were processed using the program SCRUBBER (University of Utah).

### Cell Binding Assay

Raji human Burkitt's lymphoma B-cells (American Type Culture Collection, Manassas, VA) were maintained in RPMI-1640 media supplemented with 10% fetal bovine serum, 2 mM L-glutamine, 1 mM sodium pyruvate, 1% of a solution of nonessential amino acids (GIBCO #11140–050), and 100 units/ml of Penicillin G, 100 μg/ml Streptomycin, and 0.25 μg/ml of Amphotericin B at 37°C in a humidified 5% CO_2 _atmosphere. Jurkat's cells (American Type Culture Collection, Manassas, VA), an acute leukemia T-cell line, were maintained in the same medium with the addition of 10 mM 4-(2-hydroxyethyl)-1-piperazineethanesulfonic acid (HEPES).

A series of experiments were conducted to quantify the uptake of the ^111^In-labeled parent SHAL (DvLPBaPPP)_2_LLDo and its hexa-arginine analog (DvLPBaPPP)_2_LArg_6_AcLLDo by Raji cells, a cell line that has been previously shown to express the HLA-DR10 variant. The assays were conducted using aliquots containing 10^6 ^cells suspended in 150 μl of PBS with 5% bovine serum albumin (BSA). Aliquots of cells were treated with 0.1, 1, 5, 10 or 25 ng of ^111^In-labeled (DvLPBaPPP)_2_LLDo or (DvLPBaPPP)_2_LArg_6_AcLLDo for one hour at both 4°C and 22°C. The tubes containing the treated cells were centrifuged to separate the cell pellet from the supernatant and the two fractions were counted in a calibrated gamma well counter to determine the amount of bound and free SHAL. Half of the cell pellets were washed twice with PBS and incubated at 22°C for 15 min before centrifuging them again. The pooled washes and washed cell pellets were subsequently counted in the gamma well counter to assess how much of the bound SHAL could be removed by washing.

### 3-D Confocal Microscopy

SHAL binding and internalization by Raji and Jurkat's cells was assessed using the method described previously by O'Donnell et al[48]. Experiments were conducted comparing the binding of (DvLPBaPPP)_2_LLDo (the parent SHAL), its hexa-arginine analog (DvLPBaPPP)_2_LArg_6_AcLLDo, and chimeric Lym-1 (chLym-1) to Raji cells. All steps were performed at 20°C.

Four million Raji cells (>92% viability) in log phase growth were pelleted at 300 × g, washed, and blocked for 30 min in 1 ml of 1% fraction V BSA in PBS, with constant rotation. Cells were then incubated 1 hr, at a concentration of 1 million cells per 250 μl, with either 1% BSA in PBS or a biotinylated primary reagent: 10 nM chLym-1, 10 μM parent SHAL, or 10 μM hexa-arginine SHAL. After four washes (two in 1% BSA in PBS, two in PBS), 50 μl of the cell suspensions was applied to freshly poly-L-Lysine coated slides, and cells were allowed to adhere for 10 min in a humid chamber. Fixation and permeabilization were performed at -20°C by using a 4 min exposure to methanol. Jurkat's cells were treated in the same manner as a control.

Slides were then washed twice in PBS and blocked in 10% fetaplex serum (Gemini Bioproducts, West Sacramento, CA) in PBS for 15 min and washed once in PBS. The detection reagent, Streptavidin AlexaFluor 610 (Invitrogen, Carlsbad, CA) was diluted 1/500 in diluent, 100 μl was applied; a parafilm cover slip was layered over the solution to prevent evaporation. The slides were incubated in a humid chamber for 30 min., washed 5 times for 5 min each in PBS, and rinsed briefly in double distilled H2O. After the slides dried, cover slips were mounted with ProlongGold with 4',6-diamidino-2-phenylindole (DAPI, Invitrogen, Carlsbad, CA). The slides were viewed with an Olympus FV1000 laser scanning confocal microscope and data were collected as Z-scans at 160X, with focal sections being taken 1 μm apart through the cell.

### Statistical Analysis

Data is reported as mean ± SD. Statistical comparisons were based on the Wilcoxon rank sum test [49], a procedure based on ranking the values of two test groups. Differences were considered statistically significant if p values were ≤ 0.05. The p-values were determined by the transformation Z = TANH^-1^r for the correlation coefficients [50].

## Abbreviations

Ac: acetate; Ba: N-benzoyl-L-arginyl-4-amino benzoic acid; Boc: tertiary butyloxycarbonyl; BSA: bovine serum albumin; CAE: cellulose acetate electrophoresis; CPP: cell penetrating peptide; DAPI: 4',6-diamidino-2-phenylindole; Dde: 1-(4,4-dimethyl-2,6-dixoxcyclohex-1-ylidene)ethyl; DIEA: N, N Diisopropyl-ethylamine; DMF: dimethylformamide; DMSO: dimethylsulfoxide; DOTA: 1,4,7,10-tetraazacyclododecane-1,4,7,10-tetraacetic acid; Dv: dabsylvaline; EDTA: ethylenediaminetetraacetic acid; ESI-MS: electrospray ionization mass spectrometry; Fmoc: fluorenylmethyloxy; HEPES: 4-(2-hydroxyethyl)-1-piperazineethanesulfonic acid; HPLC: high performance liquid chromatography; MeCN: acetonitrile; NHS: N-hydroxysuccinimide; Pbf: 2,2,4,6,7-Pentamethyldihydrobenzofuran-5-sulfonyl; PBS: phosphate buffered saline; PF6: hexafluorophosphate; RP-HPLC: reversed phase high performance liquid chromatography; SDS: sodium dodecylsulfate; SHAL: selective high affinity ligand; TFA: trifluoroacetic acid; TLC: thin layer chromatography.

## Competing interests

The authors declare that they have no competing interests.

## Authors' contributions

RB designed the SHAL and together with GLD planned all the experiments and interpreted the data. SH developed the synthetic scheme and synthesized the SHALs, SJD assembled and analyzed the confocal microscopy images, AN radiolabeled and purified the labeled SHAL, GM isolated and purified the HLA-DR10 protein and conducted the cell binding experiments, and MC conducted the Biacore binding experiments. All authors participated in data analysis and manuscript preparation. All authors read and approved the final manuscript.
